# Haemoglobin S and β^Thal^: Their Distribution in Maharashtra, India

**Published:** 2013-06

**Authors:** B. P. Urade

**Affiliations:** Department of Anthropology, Pune University, Pune, India

**Keywords:** haemoglobinopathies, sickle cell anemia, thalassaemia, eastern maharashtra, India

## Abstract

It has been more than six decades since the first report of sickle cell anaemia in Indian subcontinent. Since then the researchers have been reported various haemoglobin varients prevalent in India, they are HbS, Hbβ^T^, HbE and HbD. Earlier studies were confined to tribal and scheduled castes populations as if sickle haemoglobin was restricted to these two groups only. Since a decade or so, few studies on haemoglobinopathies from other Indian populations are available. Examination of premarital age group of 5172 Indian subjects (2762 males and 2410 females) from eastern Maharashtra of India showed high incidences of HbS (0-33 per cent) and Hbβ^T^ (0-10 per cent) in different ethnic groups. In present study cumulative gene frequency for HbS and Hbβ^T^ was found to be of 6.1 per cent and 2.3 per cent respectively. In present study sickle cell gene has been found in general categories of Indian populations besides scheduled castes and tribal populations. In Scheduled tribes HbS ranges from 0-24 per cent, in Scheduled castes and Nomadic tribal groups, HbS ranges from 0-13 per cent, in Other Backward caste categories it varies from 0-20 per cent while in higher caste populations it ranges from 0-5 per cent. The incidences of HbS are much higher among tribal groups than that found in other caste populations. The incidences of homozygous individuals are very few in HbS and Hbβ^T^. The hitherto regional and populations specific Hbβ^T^ haemoglobin variant in Sindhi and Bengali communities is gradually spreading in other populations of Maharashtra as evident from the present study. Lesser value of MCV, MCH and MCHC in homozygous Hbβ^T^ is due to impairments of synthesis β-globin chain. The subject with the presence of β-thalassaemia is accompanied by raised level of HbA2. Unusual higher values of RBC and WBC suggest the high concentration of hypochromic microcytosis in anemia. The means of MCV MCH and MCHC in Hbβ^T^ are much lower than the normal ranges compared to HbS.

## INTRODUCTION

Haemoglobinopathies are concerned with the abnormality in the protein molecule of red blood cells. This abnormality is due to defective synthesis of globin chains or its structure. A genetic defect that results in abnormal structure of one of the globin of the haemoglobin molecule is termed as haemoglobinopathy. These inherited genetic diseases of haemoglobin are controlled by a single gene and are transmitted from generation to the next. In India the presence of Sickle cell gene (HbS) was first detected in Nilgiri Hills of southern part (1). Sickle cell is most common pathological haemoglobin variant worldwide (2). The Indian subcontinent is a rich reservoir of sickle cell anaemia (SCA), thalassaemia (β- thal) and various abnormal haemoglobins. In India, HbS gene (‘HbS’ for HbAS carrier) is mostly confined to tribes in central and south India and the frequency ranges from 5 to 35 per cent (3) and in most of the Indian populations, castes and tribes the high incidence of various abnormal haemoglobins has been reported (4). In Central India study on sickle cell anaemia has been carried out mostly on tribal groups and very few on castes and other populations (5). WHO (2006) has reported an estimate of about 20-25 million homozygous individuals for sickle cell disease worldwide of which 5-10 million are in India (6).

β-thalassaemia (‘Hbβ^T^’ for β-thalassaemia carrier) is a major monogenic single gene disorder resulting from a reduced or absent synthesis of β-globin chain. Hbβ^T^ is most common in communities like, Sindhi, Parsee and Lohana and different ethnic groups of Punjabi, Bengali and Gujarati (7-10). In central India, the prevalence of Hbβ^T^ is high in Sidhi population (5, 9). Nowadays approximately 5per cent of the world populations are carriers of a potentially pathological haemoglobin gene (heterozygote condition). Every year about 300,000 new cases of thalassaemia syndrome (30 per cent) and sickle cell anemia (70 per cent) are detected worldwide. Globally, the percentage of carriers of thalassaemia is greater than that of carriers of SCA, but due to the higher frequency of the sickle cell gene in certain regions, the number of affected newborns is more evident. The general incidence of β-thalassaemia trait and sickle cell haemoglobinopathy varies between 0-17 per cent and 0-44 per cent, respectively. In India, as a consequence of high consanguinity, caste and area endogamy, some communities exhibits higher incidences of the diseases, what determines a major public health problem (10, 11). Earlier reports confirmed very high frequency of sickle cell trait (>20per cent) among different Indian populations (12-23).

## MATERIAL AND METHOD

A systematic mass screening was carried out in various schools and at the community level in four districts of eastern Maharashtra, India. A sample from premarital age groups of 5172 unrelated individuals, comprising 2762 males and 2410 females, were screened for haemoglobin S and haemoglobin β^T^ using solutions of qualitative solubility test and NESTROFT, respectively. Written consents were obtained from all individuals before subjecting them to the tests. A volume of 20 µl of peripheral blood samples were drawn from finger puncture for each test and mixed thoroughly with solutions. Results for haemoglobin S variant were noted after 3 minutes and for haemoglobin β-variant after 20-25 minutes. Samples with turbidity and opaque were considered positive for sickle cell and thalassaemia as per described procedure (24). Additionally, samples of intravenous blood (2 mL) were drawn in EDTA containing vacutainer tubes were used for further analysis. Haematological parameters were measured using calibrated ERMA particle counter. All the samples were subjected to haemoglobin electrophoresis using cellulose acetate membrane in alkaline TEB buffer at pH 8.9 for pattern confirmation. Control samples of HbS and Hbβ^T^ were run in electrophoresis as standards. A2 fraction of adult haemoglobin was estimated by elution method of 413 nm using spectrophotometer as well as HPCL, and the ratio of >3.5 was taken as cut off point for determination of positive for Hbβ^T^.

## RESULTS

Table 1 portrays the frequency distribution of different haemoglobin variants viz., HbA, HbS,HbSS and Hbβ^T^. It was observed that 333 individuals (6.4 per cent) were positive for HbS and 110 (2.1 per cent) for Hbβ^T^. The frequency for HbS and Hbβ^T^ varied between 0-33 per cent and 0–10 per cent, respectively, in all studied populations. A very high frequency of HbS was encountered among the Bais (33.3 per cent) the Pardeshi (25 per cent), the Pardhan (23.8 per cent) and the Marar (20.4 per cent). A moderate HbS frequency was observed among Dhiwar (12.8 per cent), Gond (12.4 per cent), Shimpi (11.1 per cent), Mahar (10.9 per cent), Madgi (10.0 per cent), Khaire kunbi (9.3), Bania (9.1 per cent) the Zade kunbi (7.7 per cent), the and the Gowari (7.3 per cent). Significant frequencies of HbS were also observed among the Banjara (5.9 per cent), the Dange kunbi (5.6 per cent), the Kunbi (4.9 per cent), the Telugu (4.8 per cent), the Kalar (4.7 per cent), the Bawane kunbi (4.3 per cent), the Brahmin (4.2 per cent), the Muslim (3.7 per cent), the Tirale kunbi (3.6 per cent) and the Teli (3.2 per cent). The range of HbS gene frequency of 1-3 per cent was accounted for the Chambhar, the Dhangar, the Dhobi, the Halba, the Kohali, the Lohar, the Maratha kunbi, the Mehetar, the Powar and the Rajput of eastern region of Maharashtra. The individuals for homozygous HbS category were very few. The results also showed a moderate frequency of β-thalassaemia (10.4 per cent) among the Sindhi. Few cases of β-thalassaemia in other tribal and caste groups have been noticed. However, in some of the sub-groups and castes no abnormality of any Hb variant was detected (Table 1). The results demonstrated that in the studied population the sickle cell trait is the most common haemoglobinopathy (6.2 per cent) followed by β-thalassaemia carrier status (2.1 per cent) and sickle cell disease (0.2 per cent).

**Table 1:** Profile of Haemoglobinopathies in eastern Maharashtra, India

Community	N°	HbAA		HbAS		HbSS		β-thal	
		M	F	No	per cent	No	per cent	No	per cent

Bais	6	2	2	2	33.3	-	-	-	-
Bania	11	8	2	1	9.1	-	-	-	-
Banjara	17	10	4	1	5.9	1	5.9	1	5.9
Bawane kunbi	23	11	11	1	4.3	-	-	-	-
Beldar	21	13	8	-	-	-	-	-	-
Bengali	12	9	3	-	-	-	-	-	-
Brahmin	144	89	49	6	4.2	-	-	-	-
Chambhar	52	28	25	1	1.9	-	-	-	-
Christian	19	13	6	-	-	-	-	-	-
Dange kunbi	18	8	9	1	5.6	-	-	-	-
Dhangar	36	20	14	1	2.7	-	-	1	2.7
Dhanoje kunbi	2	-	2	-	-	-	-	-	-
Dhiwar	39	17	17	5	12.8	-	-	-	-
Dhobi	34	19	12	1	2.9	-	-	2	5.9
Gond	218	101	89	27	12.4	-	-	1	0.4
Gowari	55	29	22	4	7.3	-	-	-	-
Gujarati	13	6	7	-	-	-	-	-	-
Halba	138	86	49	2	1.4	-	-	1	0.7
Kalar	106	52	49	5	4.7	-	-	-	-
Katia	2	-	1	1	50	-	-	-	-
Khaire kunbi	246	94	129	23	9.3	-	-	-	-
Khatik	7	4	3	-	-	-	-	-	-
Kohali	37	16	20	1	2.7	-	-	-	-
Kumbhar	16	7	9	-	-	-	-	-	-
Kunbi	184	77	98	9	4.9	-	-	-	-
Lewa kunbi	4	1	3	-	-	-	-	-	-
Lohar	47	22	24	1	2.1	-	-	-	-
Lonare kunbi	3	2	1	-	-	-	-	-	-
Madgi	40	21	15	4	10	-	-	-	-
Mahar	1,551	721	652	170	11	8	0.5	-	-
Marar/Mali	93	31	43	19	20.4	-	-	-	-
Maratha kunbi	46	15	30	1	2.2	-	-	-	-
Marwadi	12	10	2	-	-	-	-	-	-
Matang	3	1	2	-	-	-	-	-	-
Mehetar	62	29	32	1	1.6	-	-	-	-
Mochi	16	11	4	1	6.3	-	-	-	-
Muslim	160	116	38	6	3.8	-	-	-	-
Pardeshi	4	1	2	1	25	-	-	-	-
Pardhan	21	10	5	5	23.8	1	4.7	-	-
Powar	112	54	52	2	1.8	-	-	4	3.5
Rajput	48	28	18	1	2.1	-	-	1	2.1
Shimpi	9	5	3	1	11.1	-	-	-	-
Sikh	28	23	4	-	-	-	-	1	3.6
Sindhi	931	408	424	2	0.2	-	-	97	10.4
Sutar	26	13	13	-	-	-	-	-	-
Teli	313	170	133	10	3.2	-	-	-	-
Telugu	21	15	5	1	4.8	-	-	-	-
Tirale kunbi	110	68	37	4	3.6	-	-	1	0.9
Walmiki	4	1	3	-	-	-	-	-	-
Yadav	39	31	8	-	-	-	-	-	-
Zade kunbi	13	7	5	1	7.7	-	-	-	-
Total	5172	2762	2410	323	6.2	10	0.2	110	2.1

Table 2 demonstrates the red cell morphology for HbS and Hbβ^T^. From the table it has been observed that carrier HbS pooled data show higher mean values of WBC, HGB, MCV, MCH, MCHC and RDW but lower values of RBC and PLT higher than those for Hbβ^T^.

**Table 2:** Mean ± SD values of red cell morphology among carriers of HbS and Hbβ^T^ individuals

HbVariant	Sex	No	Mean	WBC	RBC	HGB	HCT	MCV	MCH	MCHC	RDW	PLT

HbAS	M	115	M	9.1	6.5	12.4	48.7	75.5	20.7	27.75	14.1	417.3
			SD	3.1	2.3	2.3	16.4	14.9	6.1	9.0	2.6	284.2
	F	78	M	9.4	5.9	11.8	45.2	78.1	23.4	28.4	15.0	385.3
			SD	3.2	2.0	1.9	13.9	14.9	13.8	10.5	2.7	162.8
Hbβ^T^	M	52	M	8.6	8.1	12.1	50.6	63.8	15.8	25.3	12.9	457.4
			SD	3.0	2.9	2.2	17.4	11.3	3.7	4.9	1.7	246.4
	F	58	M	8.4	7.1	10.8	43.8	61.7	15.9	25.9	12.6	446.5
			SD	2.9	2.2	1.8	14.1	8.9	3.6	5.7	1.9	208.7

## DISCUSSION

Haemoglobinopathies are the most common monogenic disorder affecting millions of people worldwide. The geographical distribution of HbS and Hbβ^T^ variants in India is not uniform as the prevalence varies in different regions of the country from 0–44 and 1-15 per cent respectively. In central India the incidence of β-thalassaemia has been mainly attributed to its high prevalence in the migrant population of Sindhi origin. Screening of healthy population is required to determine the carrier rates and gene frequencies in this region. Because of the complications associated with haemoglobinopathies and frequent health crisis, these genetic disorders are becoming a growing health care problem in all regions of the developing countries.

The high incidences of HbS gene in tribes than in castes attribute the age old practices of consanguinity among them (25). Earlier researchers have stigmatized the tribal and low caste populations with prejudice mind of confining sickle cell gene with these groups. Wrong notions that were deeply rooted in the Indian society are that the high incidence of sickle cell gene among the tribes and lower castes is due to admixture (12, 13, 26, 38). On the contrary, as evident from the present study the occurrence of sickle cell gene among higher caste populations is itself an indication of carrying this mutant gene from ancient times independently. It is needless to say that SCA is confined to the lower caste and tribal groups only. Sickle cell gene in India was probably brought by some high caste populations from Africa through different route, which subsequently, penetrated the scheduled castes and then tribes (11). The presence of HbS among higher communities pave the way of transmitting this gene in to lower castes during the ancient period as the higher caste people used to exploit socially and economically backward populations (5). On the contrary, the sickle cell anaemia has got its unicentric origin of the mutation in India and unicentric origin of tribal population in India (27). Moreover, the sickle cell is not necessarily a tribal trait (28). Sickle cell anaemia is not only confined to the lower castes or tribal groups but it is equally prevalent significantly among higher caste populations too. The low frequency of homozygous S (HbSS) may be due to early death during infant life without knowing reason or many of them go unnoticed because of lack of awareness of the disease.

Earlier studies demonstrated a complete absence of HbS gene in Brahmin (29) Muslim (30, 31) and Dhangar (32), among these three caste groups the incidences of sickle cell gene varies from 2-4 per cent, this finding is in conformity with earlier studies (33, 34). Hitherto unreported sickle cell gene in Rajput and Kalar also possess sickle cell gene in these endogamous groups with a range of 2-5 per cent. The high frequency among the Pardhan demonstrated in this study was reported earlier by other groups (26, 35). Among the Mahar of central India the frequency of HbS ranges from 0 to 24 per cent while that of sickle cell disease ranges from 0 to 6 per cent (5, 13, 25, 37, 39, 40, 41, 42). The present findings show a moderate frequency of HbS among the Mahar and the Kunbi, thus differ in agreement with the results that reported a very high frequency of HbS among these two endogamous groups. Earlier studies from similar environmental areas were conducted by some researchers show different frequencies in the occurrence of sickle cell anaemia among Mahar. In late 50’s the frequency of sickle cell anaemia in Nagpur city was 22.22 per cent (42) in early 60’s it was reduced 18.05 per cent (13) while in 80’s it was 18. 75 per cent (43) and in 2012 it was 11.81 per cent (5). This situation suggests that education and health awareness may be attributed to declining in the occurrence of sickle cell anaemia among the Mahar with the increase education level. The present study confirmed that sickle cell disease is prevalent in Maharashtra, Madya Pradesh, Orissa, Andhra Pradesh, Gujarat, Tamil Nadu, Karnataka, Kerala and Uttar Pradesh (34). Earlier study has reported very high frequency of Hbβ^T^ gene among the Sindhi (9). However, the present study has recorded a moderate frequency among Sindhi and low among few ethnic groups is the indication of either gradual spread of β-gene due to hybridization or due to independent mutation which invites in-depth research to conclude this phenomenon.

The subdivision of the population in India by geographic, linguistic, religious and caste barriers has resulted in the existence and perpetuation of thousands of distinct highly inbred communities (20). This remarkable genetic heterogeneity is a distinctive feature of the Indian population that can contribute to a variable distribution of the haemoglobinopathies. The diffusion of HbS mutation from the Middle East region to India by Arab and Muslim expansion was proposed (36). But in most of the cases, HbS mutation in the Middle East and in India shares a common haplotype as such the higher prevalence of HbS among the tribal and scheduled caste populations of India does not reinforce this hypothesis.

The spread of Hbβ^T^ gene in the region is probably due to gene flow or people migration from north-west pre-independent India (ie, Sindh region of Pakistan) (5) as similar to those found among some ethnic groups of West Bengal due to Portugalete’s. The present findings confirm that β haemoglobin has high frequency in the region from where they had spread to other parts of the central and western India. It is also interesting to note that higher frequencies of abnormal haemoglobins S in southern, central, western and parts of eastern India is due to castes and area endogamy.

The red cell count was relatively higher in relation to the haemoglobin, MCH and MCHC in Hbβ^T^ carrier than that found in HbS carrier, the situation leads to common feature of iron deficiency in Hbβ^T^ carrier individuals. Lower value of WBC and RDW accompanied by higher RBC in β-thalassaemia carrier encountered with hypochromic and microcytoic red cell indices.

## CONCLUSION

The people of India are culturally as well as biologically stratified in to various ethnic groups. In this study the presence of HbS gene in almost all castes and communities demonstrate that sickle cell anemia is no longer confined to specific ethnic groups; instead it is widely distributed in all tribal, scheduled caste, backward and higher caste native in this region. Similarly, sporadic occurrence of Hbβ^T^ in the present study suggests the spread of Hbβ^T^ well beyond the Sindh and Punjab regions to central India. The high magnitude of HbS and β^T^ appears to contribute significantly to the frequency of haemoglobinopathies in this region. It is important to state that the presence of HbS and β^T^ in previews unreported communities from this region is due to a lack of research, under-diagnosis, and absence of proper diagnostic facilities at prenatal stage.

The most effective approach to minimize the problem of haemoglobinopathies in India is to offer genetic counseling, proper health education, sensitization to the individual concern, prenatal diagnosis and selective termination of pregnancy of the affected fetus. Additionally, some strategies should be implemented in the prevention and in the management of the disease.
